# Screening history and risk of death from prostate cancer: a nested case–control study within the screening arm of the Finnish Randomized Study of Screening for Prostate Cancer (FinRSPC)

**DOI:** 10.1007/s10552-023-01828-2

**Published:** 2023-12-08

**Authors:** Kirsi Talala, Stephen Walter, Kimmo Taari, Teuvo L. J. Tammela, Paula Kujala, Anssi Auvinen

**Affiliations:** 1 Finnish Cancer Registry, Unioninkatu 22, 00130 Helsinki, Finland; 2 Faculty of Health Sciences, McMaster University, Hamilton, ON L8S 3L8 Canada; 3 Department of Urology, Helsinki University Hospital and University of Helsinki, 00029 Helsinki, Finland; 4 Faculty of Medicine and Health Technology, Tampere University, 33014 Tampere, Finland; 5 Department of Surgery, Tampere University Hospital, 33521 Tampere, Finland; 6 Department of Pathology, Fimlab Laboratories, 33101 Tampere, Finland; 7 Faculty of Social Sciences/Health Sciences, Tampere University, 33014 Tampere, Finland

**Keywords:** Prostate cancer, Screening, Case–control study, Mortality

## Abstract

**Purpose:**

We assessed the risk of death from prostate cancer (PCa) in relation to men’s screening histories, i.e., screening attendance among men who were offered screening.

**Methods:**

Men in the Finnish Randomized Study of Screening for Prostate Cancer (FinRSPC) screening arm were invited to up to three screening rounds with the serum prostate-specific antigen (PSA) test at 4-year intervals during 1996–2007.

Case subjects (*n* = 330) were men who died from PCa. Each case was matched to five controls (*n* = 1544) among the men who were free of PCa.

Screening history was defined as (1) never/ever attended screening prior to the case diagnosis; (2) attended at the first screening round; and (3) recency of screening, calculated as the time from last screening attendance to the date of case diagnosis.

The association between screening history and the risk of death from PCa was estimated by odds ratios (OR) with 95% confidence intervals (CI) using conditional logistic regression.

**Results:**

Having ever attended screening versus never attended was associated with a reduced risk of PCa death (OR 0.60, 95% CI 0.45–0.81) and a similar association was found for those attended (versus not attended) the first screening round (OR 0.67, 95% CI 0.51–0.87).

The effect by time since last screen for the risk of PCa death was significantly lower 2–7 years since last screen.

**Conclusion:**

Among men invited to screening, subjects who attended any PSA screening during the previous 19 years had a 40% reduction in PCa mortality compared to non-screened men.

## Introduction

Cancer screening is intended to reduce cancer mortality from the disease of interest by advancing the time of diagnosis, through detection of asymptomatic disease at an earlier stage, compared to when diagnosis would occur if screening had not taken place. The European Randomized Study of Screening for Prostate Cancer (ERSPC), which has been conducted in eight European countries, is intended to assess the impact of screening using prostate-specific antigen (PSA) [1]. Its most recent multi-center analyses showed reduced incidence of advanced PCa disease [2], and a 20% relative reduction in PCa mortality over 16-year follow-up [3]. In the Finnish section of the ERSPC (FinRSPC) alone, there was a non-significant 10–11% reduction in prostate cancer mortality at 14 and 15-year follow-up [4, 5].

In the ERSPC and FinRSPC trial, we previously evaluated the impact of offering screening compared to controls not offered screening. An intention-to-treat analysis was employed, comparing groups defined by random allocation, which allows for possible non-compliance with screening in the screening arm, and for contamination of the control arm, where no intervention was offered within the trial. This type of analysis assesses the benefit of a screening program in a population, and advises public policy concerning the effectiveness of the screening [6].

In contrast, with a case–control design, different perspectives can be used to evaluate cancer screening. The benefit assessed in case–control studies is one of efficacy of screening tests [7, 8]. The efficacy measures are appropriate for individual decision-making and assess the benefit of screening among subjects who are actually screened within the screening arm [8]. Signal et al. [9] defined efficacy as the performance of an intervention under ideal and controlled circumstances compared to effectiveness, which refers to the performance of an intervention under ‘real-world' conditions. Therefore, efficacy analysis maximizes the likelihood of observing an effect of the screening intervention if one indeed exists. This is valuable information for individuals about the benefits of attending screening.

Our aim was to evaluate the efficacy of screening, by estimating the risk of prostate cancer death associated with various screening histories, i.e., with screening attendance profiles among men who were offered screening.

## Methods

### Setting and source population

The study used data from the population-based Finnish Randomized Study of Screening for Prostate Cancer (FinRSPC), which is the largest component of the multi-center European Randomized Study of Screening for Prostate Cancer (ERSPC) trial. The Finnish trial recruited 80,458 men during 1996–1999 at two screening centers, in Helsinki and Tampere. During 1996–1999, 8,000 men residing in the metropolitan areas of Helsinki and Tampere aged 55, 59, 63 or 67 years were randomized each year to the screening arm (*n* = 32,000), or to the control arm (*n* = 48,458). Exclusions due to before randomization PCa diagnosis, death or emigration from the study area were in total *n* = 134. Due to logistical reasons, 1,667 eligible men in the screening arm were not invited for the first screening round. The remaining *n* = 30,199 men in the screening arm were invited to be screened using the serum prostate-specific antigen (PSA) test for three screening rounds at four-year intervals until 2007. Of the men invited in the screening arm, 78.7% attended and were actually screened at least once (*n* = 23,771). The trial protocol has been described previously in detail [4, 10].

### Selection of cases

**Fig. 1:**
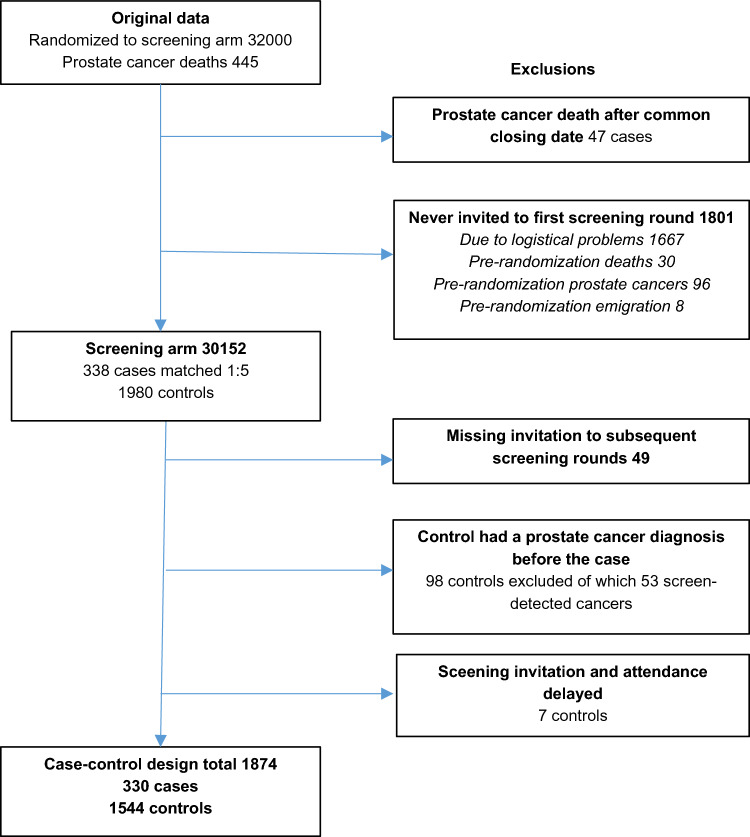
Study flow chart

The present study was designed as a nested case–control study within the FinRSPC trial (Fig. 1). Case subjects were men from the screening arm who died from PCa between trial entry and the end of follow-up (31 December 2017). All case subjects were required to be invited for their first screening tests before being diagnosed with PCa. The underlying cause of death (code C61 in ICD-10) was identified through Statistics Finland (research permission TK/3536/07.03.00/2021). Within the trial, the cause of death was additionally reviewed by an adjudication committee for all deaths among men with prostate cancer during 1996–2003, and the results showed excellent agreement (97.7%; *κ* = 0.95) with the official statistics [11].

Each participant entered study on January 1 in their year of randomization (1996–1999), and follow-up ended at death, emigration, or the common closure date for the study analysis (31 December 2017). Follow-up was truncated after a maximum of 19 years for each man.

### Selection of controls

Controls were selected—as cases—among men from the trial screening arm, using incidence density sampling with replacement. This involves choosing control subjects among men at risk of disease and eligible for screening, i.e., who were alive at the time of the death of their matched case, and free of prostate cancer until the date of the corresponding case diagnosis. Each case was matched to five controls on year of birth. Controls were given a pseudo-diagnosis date equal to the diagnosis date of the matched case subject. Restricting the screening history of the control subjects to before the pseudo-diagnosis date prevents control subjects from having a greater screening opportunity than their matched case subject. In particular, to ensure comparability between cases and controls,—similar to cases—, follow-up started at the time of randomization, and any screening experience after the date of diagnosis of the case was censored [12, 13]. However, men were randomized by birthyear to be invited for screening during the same calendar year at random times. To avoid the possibility that screen-detected cases had been invited, tested, and diagnosed before their randomized control subjects had been invited for that same calendar year, we included control screens (*n* = 178) after the date of diagnosis that took place during the same invitation year as for cases.

Our data thus comprised of 330 cases aged 58–84 years at the time of their death from prostate cancer and 1,544 matched controls.

### Secondary sample

As a secondary analysis, we also drew an intention-to-treat trial sample applying similar criteria as in the primary FinRSPC analysis, but with cases and controls sampled from both the screening and control arms of the trial. This approach was used to obtain effectiveness estimates comparable to those reported in the original analysis [7], in order to assess whether our sampling of control subjects from the screening arm only might have introduced bias.

### Screening history measure definitions

For the analyses, screening history was measured in (1) never/ever screened in a given period of time before the case subject had been diagnosed, and (2) screening attendance in response to the first screening invitation, and (3) recency, defined as the time between the case diagnosis and the most recent screening test. The ever/never measure is the recommended exposure for estimating screening efficacy [7]. For the cases and their matched controls, screening attendance was defined as having any screening visit in the period from entry into the trial (randomization) up to the year of the case diagnosis. We considered screening year rather than exact date to allow equal opportunity to be screened for both cases and controls who were randomly invited the same year.

### Statistical analysis

The association between screening history and the risk of death from PCa was estimated by odds ratios (OR) with their 95% confidence intervals (CI) using conditional logistic regression, which is appropriate and a standard method for matched sets. Conditional (fixed-effects) logistic regression stratifies the analyses by case–control strata and takes matching into account. We evaluated time since last screening test until diagnosis. All *p* values were obtained from two-sided statistical tests, with *p* < 0.05 considered statistically significant. All analyses were performed with STATA (version 14, StataCorp) [14].

Potential confounding factors were considered, such as educational level and socioeconomic group obtained from Statistics Finland, self-reported family history of prostate cancer, and comorbidity (Charlson Comorbidity Index, CCI), which was calculated by using the pooled diagnostic data from hospitalization data (ICD-10 from the Finnish national Care Register for Health Care, HILMO) and the medication databases of the Social Insurance Institution of Finland (SII) [15]. These factors could be related to screening frequency and also potentially increase the risk of prostate cancer mortality (Table 1). Based on our preliminary analysis, socioeconomic group and family history did not appear to be important confounders in our data, and they were therefore excluded as covariates. Neither showed important associations with prostate cancer death, as we have also reported previously in detail for family history [16]. Final models were adjusted for educational level and comorbidity.

**Table 1:** Background characteristics of the case subjects and the controls, total *n* = 1,874

	Cases *n* = 330	Controls *n* = 1,544
Mean age at diagnosis of case (years)	68.5 (54.5–84)	68.2 (54.4–84.1)
Mean follow-up time (years)	12.7 (2.1–19)	12.6 (2.1–19)
Educational level (%)		
Highest	28.2	34.8
Intermediate	20.3	21
Lowest	51.5	44.2
Socioeconomic group (%)		
Upper-level employee	12.7	14.8
Lower-level employee	8.8	9.7
Self-employed	3	7.1
Manual worker	11.5	8.8
Retired	50	47.3
Unemployed	12.1	10.1
Not known	1.8	2.2
Family history for PCa (%)	8.2	7.8
Co-morbidities (%)		
0	77.2	88.9
1	9.5	7.6
2–4	13.2	3.5
Number of screening invitations (%)		
1	44.5	44.2
2	35.5	33.6
3	20	22.2
Number of screens (%)		
0	26.7	17.4
1	39.4	38.1
2	25.8	29.3
3	8.2	15.3
Mean number of screening rounds (excluding never screened)	1.6	1.7

A never/ever odds ratio was calculated for men having had any screening test within the 19-year follow-up period, compared to having had no screening test. We used a range of intervals in separate multivariate models, in order to evaluate a range of time windows (1–19 years) in each analysis, and thereby define a relevant time period for defining never/ever screened, to reflect a time period when disease might or might not be detected. The odds ratio for the first screening test was calculated for having versus not having attended at the first screening round (Table 2).

**Table 2:** An overall odds ratio and range of intervals in separate conditional logistic regression models with different exposure window for having had screening test within the 19-year period as compared with no screening test (never/ever)

	Cases	Controls	OR	95% CI	OR^b^	95% CI^b^
Ever screened vs. never screened						
Never screened	88	268	1			
Ever screened	242	1,276	**0.58**	**0.44–0.77**	**0.60**	**0.45–0.81**
First screen attender vs. non-attender						
Non-attender	107	359	1			
First screen attender	223	1,185	**0.64**	**0.49–0.83**	**0.67**	**0.51–0.87**
Screened within 1 year before^a^ vs. not screened						
Not screened	215	994	1			
Screened	115	550	1.05	0.72–1.54	1.04	0.69–1.55
Screened within 2 years before^a^ vs. not screened						
Not screened	203	869	1			
Screened	127	675	0.73	0.52–1.02	0.72	0.51–1.03
Screened within 3 years before^a^ vs. not screened						
Not screened	193	767	1			
Screened	137	777	**0.60**	**0.44–0.82**	**0.62**	**0.45–0.85**
Screened within 4 years before^a^ vs. not screened						
Not screened	169	673	**1**			
Screened	161	871	**0.67**	**0.49–0.90**	**0.70**	**0.51–0.96**
Screened within 5 years before^a^ vs. not screened						
Not screened	153	606	**1**			
Screened	177	938	**0.69**	**0.51–0.94**	**0.73**	**0.53–0.99**
Screened within 6 years before^a^ vs. not screened						
Not screened	148	526	1			
Screened	182	1,018	**0.57**	**0.43–0.76**	**0.60**	**0.45–0.81**
Screened within 7 years before^a^ vs. not screened						
Not screened	139	477	1			
Screened	191	1,067	**0.57**	**0.43–0.75**	**0.59**	**0.44–0.79**
Screened within 8 years before^a^ vs. not screened						
Not screened	129	428	1			
Screened	201	1,116	**0.56**	**0.43–0.74**	**0.59**	**0.45–0.79**
Screened within 9 years before^a^ vs. not screened						
Not screened	123	393				
Screened	207	1,151	**0.55**	**0.42–0.72**	**0.57**	**0.43–0.75**
Screened within 10 years before^a^ vs. not screened						
Not screened	115	363	1			
Screened	215	1,181	**0.56**	**0.43–0.73**	**0.58**	**0.43–0.77**
Screened within 11–12 years before^a^ vs. not screened						
Not screened	101	322	1			
Screened	229	1,222	**0.60**	**0.46–0.79**	**0.62**	**0.47–0.82**
Screened within 13–19 years before vs. not screened						
Not screened	91	280	1			
Screened	239	1,264	**0.59**	**0.44–0.77**	**0.61**	**0.46–0.82**

Time since PSA test to PCa diagnosis of the case subject associated with prostate cancer (PCa) mortality. Any screening experience after the case diagnosis year was censored. Values in bold indicate statistical significance

^a^Time to PCa diagnosis

^b^Adjusted for educational level and comorbidity

When we used recency as the screening history measure, we obtained estimates from a single conditional logistic regression model, with the 19-year follow-up period divided into intervals (< 1, 2–4, 5–7, 8–10, 11–13, 14–19 years). The odds ratios were calculated for having one's last screening test during each interval, with the reference group being persons who had no screening tests at all during the 19-year period (Table 3). The pattern of the effect by time since last screen reflects how long the risk of prostate cancer death remains different for those choosing to be screened compared to those who were never screened.

**Table 3:** Odds ratios for single conditional logistic regression model for having one’s most recent screening test during each time segment in case subjects and controls before the diagnosis of the PCa of the case subjects

	Cases	Controls	OR	95% CI	OR^b^	95% CI^b^
Not screened	88	268	1		1	
Screened 1 year before^a^	115	550	0.80	0.54–1.19	0.80	0.53–1.21
Screened 2–4 years before^a^	47	321	**0.40**	**0.26–0.62**	**0.46**	**0.29–0.71**
Screened 5–7 years before^a^	30	196	**0.40**	**0.24–0.70**	**0.42**	**0.25–0.73**
Screened 8–10 years before^a^	23	115	0.59	0.32–1.10	0.59	0.32–1.10
Screened 11–13 years before^a^	18	73	0.75	0.35–1.62	0.79	0.36–1.74
Screened 14–19 years before^a^	9	21	1.27	0.48–3.35	1.26	0.47–3.36

^a^Time to PCa diagnosis

^b^Adjusted for educational level and comorbidity. Values in bold indicate statistical significance

To explore possible confounding and effect modification by age, we conducted an additional stratified analysis comparing age < 65 years at the last screening versus ≥ 65 years. For those not attending and without a screening date, we used the date of their invitation to calculate age at last screening.

## Results

For the case–control analyses, our data comprised of 330 cases aged 58–84 years at the time of death from prostate cancer and 1,544 matched controls. The mean follow-up time was 12.7 years for the cases and 12.6 years for the controls.

Lower education, a family history of prostate cancer and comorbidities were more common among the case subjects than controls. Never attendance was more common among cases, and controls were more likely to be screened two or three times compared to cases (Table 1).

Never vs. ever screened in period of time before the case subject was diagnosed was associated with reduced prostate cancer mortality (OR 0.60, 95% CI 0.45–0.81) (Table 2). Screening attendance appeared to be associated with a constant mortality reduction as the time window for screening before diagnosis increased.

Being screened in response to the first screening round invitation (first screen attender vs. non-attender) was associated with a similarly reduced prostate cancer mortality (OR 0.67, 95% CI 0.51–0.87) (Table 2).

In the analysis of recency, where we obtained estimates for time intervals from a single conditional logistic regression model having never attended screening as a reference group, the most recent screening attendance prior to the case diagnosis to diagnosis (< 1 years including cases detected by screening), was associated with a non-significant prostate cancer mortality reduction (OR 0.80, 95% 0.53–1.21) (Table 3). The most recent screening attendance 2–4 years (OR 0.46, 95% 0.29–0.71), and 5–7 years (OR 0.42, 95% CI 0.25–0.73) before the diagnosis of the fatal PCa in the case subject were also associated with statistically significant mortality reductions. With an interval longer than 8 years since the last screen, mortality reduction attenuated.

To explore possible confounding and effect modification by age, we conducted an age-stratified analysis. Compared to the main analysis with OR 0.58; 95% CI 0.44–0.77 (Table 2) for ‘screening never vs. ever’ the result for the age group < 65 years was OR 0.55, 95% CI 0.36–0.84 and for ages ≥ 65 years OR 0.53, 95% CI 0.34–0.81. We also conducted analyses adjusting for ‘age at last screening’ on ‘screening never vs. ever’ (adjusted OR 0.60 95% CI 0.45–0.80) with minor effect, and also with no effect modification with ‘age at last screening’ in the model (likelihood-ratio test *p* = 0.64).

Similarly, we also stratified analysis for the ‘First screen attender vs. non-attender’ (OR 0.64; 95% CI 0.49–0.83, Table 2) and found OR 0.60, 95% CI 0.40–0.90 for the age group < 65 years and OR 0.55, 95% CI 0.38–0.81 for the ages ≥ 65 years. Again, adjustment for the ‘age at last screening’ showed no impact and therefore no major confounding for ‘First screen attender vs. non-attender’ (adjusted OR 0.65; 95% CI 0.50–0.84) nor effect modification (likelihood-ratio test *p* = 0.57).

Sensitivity analyses with the secondary sample with comparison of the screening and control arms using an intention-to-treat approach were conducted to examine if the estimated effects from case–control analyses were similar to the trial results in previous analyses (data not shown). With 19-year follow-up (mean 15.7 years, SD 5.3, median 19 years) prostate cancer mortality in men invited to screening was 7% lower compared to men who had not been invited to screening (OR 0.93, 95% CI 0.81–1.06). The estimate is congruent with Pakarainen et al. [5] who found a 10% mortality reduction in intention-to-treat analyses (HR 0.90, 95% CI 0.77–1.06) and an 8% reduction in a complementary case–control analysis (OR 0.92, 95% CI 0.77–1.09) at 15-year follow-up.

## Discussion

We evaluated the efficacy of prostate screening in reducing the risk of death from prostate cancer associated with screening attendance, among men who were offered screening. The results suggested that screened men were 40% less likely to die from the prostate cancer compared to men invited for screening but who did not attend. We also examined the impact of the length of time since the most recent PSA-test in reducing this risk. The effect by time since last screen for the risk of prostate cancer death was significantly lower 2–7 years since last screen compared to those who were not screened.

We analyzed a range of intervals to give a sensitivity analysis associated with the exposure window comparison of ever versus never screened and calculated the odds ratios for having a screening test during each period in separate models. This was to evaluate the duration of screening effect, after which the mortality effect would wane and approach that in non-screened men. Comparison of ever versus never screened men gave roughly constant effects of participation for a different range of screening time windows before diagnosis.

However, we found that the greatest benefit associated with screening attendance was not in the interval < 1 year to diagnosis, representing cases detected in screening. A case is eligible to be screened until cancer is detected, whereas time-matched controls are eligible to be screened only until the time of cancer diagnosis of their matched case (pseudo-diagnosis date). Therefore, when the matched case is screen-detected this design is subject to screening opportunity bias against screening [17]. PSA screening precedes < 1 year to diagnosis in screen-detected prostate cancer case subjects if the test leading to a diagnosis is included. Inclusion of the diagnostic screen increases the calculated screening rate among screen-detected cases. Exclusion of the diagnostic tests would be equivalent to counting only negative screening test results and would result in a bias in favor of screening. However, we tried to minimize the screening opportunity bias and allowed screening time to cover the corresponding screening invitation year for controls even after pseudo-diagnosis date, as cases and controls were randomized by birth year to be screened during the same calendar year. This was to avoid the situation where a case is diagnosed before the control subjects are invited to be screened for that screening year (*n* = 178).

Our study has some limitations due to the non-randomized approach. The case–control design comparing screening participants and non-participants is subject to several biases. Self-selection is due to compliance or non-compliance with screening invitations, for reasons that are related to both the likelihood of screening participation and the risk of dying from prostate cancer [18]. Studies have demonstrated the importance of self-selection adjustment [19]. To control confounding by self-selection, we made adjustment for educational level and comorbidity, which are factors that could underlie self-selection bias. Socioeconomic group and family history, on the other hand, were not used as covariates, as they were not important predictors of prostate cancer death in preliminary analyses. Family history data was obtained using a self-reported questionnaire, which may have affected its validity. Additionally, the discriminative value of the socioeconomic group measure was diminished in our data since 40% of the men were retired at the time of entry into the study. However, we used educational level, which is a fundamental and fairly stable component of socioeconomic status, and a useful measure also for those who are not in the work force. Previously we have shown that Charlson Comorbidity Index (CCI) adequately predicts mortality. However, its’ ability to discriminate men at higher risk of death was limited and diminished with longer follow-up [15].

Previous ERSPC studies found that PSA screening effectively detects all larger, more aggressive tumors in the first (prevalence) screening round. The detected tumors had significantly more favorable disease stage and grade distributions on repeat (incidence) screening [20, 21]. In our study, the first screen attenders had 33% lower prostate cancer mortality compared to never-attenders. This indicates that prostate cancers detected at the first screen were in a curable stage. We have previously found in the original intention-to-treat analyses non-significantly lower PCa mortality among the first screen invitation attenders compared to the trial control arm never invited to screening (HR 0.52, 95% CI 0.24–1.13) [5]. Intention-to-treat analyses also showed 1.25-fold PCa mortality in non-attenders than in the control arm at the first screening round. Over the total follow-up period, compared to the trial control arm who were never invited to screening, PCa mortality was 1.58-fold among those who did not attend any screening rounds (never-attender) and 1.68-fold in only one-time attenders in the screening arm. The mortality risk reduction in PCa was seen only in men attending 2–3 times. Also, all-cause mortality was higher in the never-attenders compared to the control arm (non-invited) population. Never-attenders had 1.7-fold all-cause mortality, whereas those who attended only once had all-cause mortality close to the control arm [5]. This indicates that never attenders, which we used as a reference group in the present study, includes selected men with higher rates of PCa or all-cause mortality, therefore, men with health problems, which may also affect their ability to attend screening. This may also indicate “healthy screenee bias,” in which healthy men have the opportunity to attend more screening rounds than those men with comorbidities and/or prostate cancer. Also, some of the effect we see with time since last PSA test may be due to generally healthier men having better survival after PSA testing.

ERSPC multi-center analyses showed a 20% relative reduction in PCa mortality over 16-year follow-up [3]. In the Finnish center, there was only a non-significant 10–11% reduction [4, 5], and a significant reduction in PCa mortality was found in the Swedish and Dutch centers only. Differences between centers are assumed due to differences in length of follow-up, performance of screening and duration of intervention, underlying incidence and mortality, as well as contamination in the control group [1].

The pattern of the effect by time since last screen should reflect how long the risk of prostate cancer death remains reduced for those choosing to be screened compared to those choosing not to be screened. We examined the length of the period after screening, during which the risk of fatal prostate cancer was decreased. Our data indicate that the risk of prostate cancer mortality was markedly lower for up to 7 years after screening attendance. This falls within the range described in earlier studies for the lead-time associated with prostate cancer screening. Among screen-detected cancers that would have been diagnosed in the patients’ lifetimes, the estimated mean lead-time ranged from 5.4 to 6.9 years across models [22].

Our sensitivity analyses with a secondary sample using intention-to-treat approach (comparing the trial arms) gave results similar to earlier analyses, confirming that the estimates from the case–control analysis were not biased by our sampling procedure. As expected, screening participation was associated with a larger mortality reduction (40% vs. 7%) than allocation to the screening arm in the intention-to-treat analyses, in which non-attendance in the intervention arm and opportunistic screening in the control arm dilute the association.

We assessed the efficacy of screening, i.e., benefit of attending screening when screening is offered, within a case–control setting. Our efficacy analyses showed a larger effect of screening participation on prostate cancer mortality compared to simply being randomized to the screening arm in the intention-to-treat analyses. Our present analysis will broaden the perspective and give additional information of the potential benefits for men considering attending PSA screening test when screening is offered.

## Data Availability

The datasets generated during and/or analyzed during the current study are not publicly available due to national law and data permit requirements.
